# Targeting the Mitotic Catastrophe Signaling Pathway in Cancer

**DOI:** 10.1155/2015/146282

**Published:** 2015-09-27

**Authors:** Margaret M. Mc Gee

**Affiliations:** School of Biomolecular and Biomedical Science, Conway Institute, University College Dublin, Belfield, Dublin 4, Ireland

## Abstract

Mitotic catastrophe, as defined in 2012 by the International Nomenclature Committee on Cell Death, is a *bona fide* intrinsic oncosuppressive mechanism that senses mitotic failure and responds by driving a cell to an irreversible antiproliferative fate of death or senescence. Thus, failed mitotic catastrophe can promote the unrestrained growth of defective cells, thereby representing a major gateway to tumour development. Furthermore, the activation of mitotic catastrophe offers significant therapeutic advantage which has been exploited in the action of conventional and targeted anticancer agents. Yet, despite its importance in tumour prevention and treatment, the molecular mechanism of mitotic catastrophe is not well understood. A better understanding of the signals that determine cell fate following failed or defective mitosis will reveal new opportunities to selectively target and enhance the programme for therapeutic benefit and reveal biomarkers to predict patient response. This review is focused on the molecular mechanism of mitotic catastrophe induction and signalling and highlights current strategies to exploit the process in cancer therapy.

## 1. Introduction 

**Figure 1:**
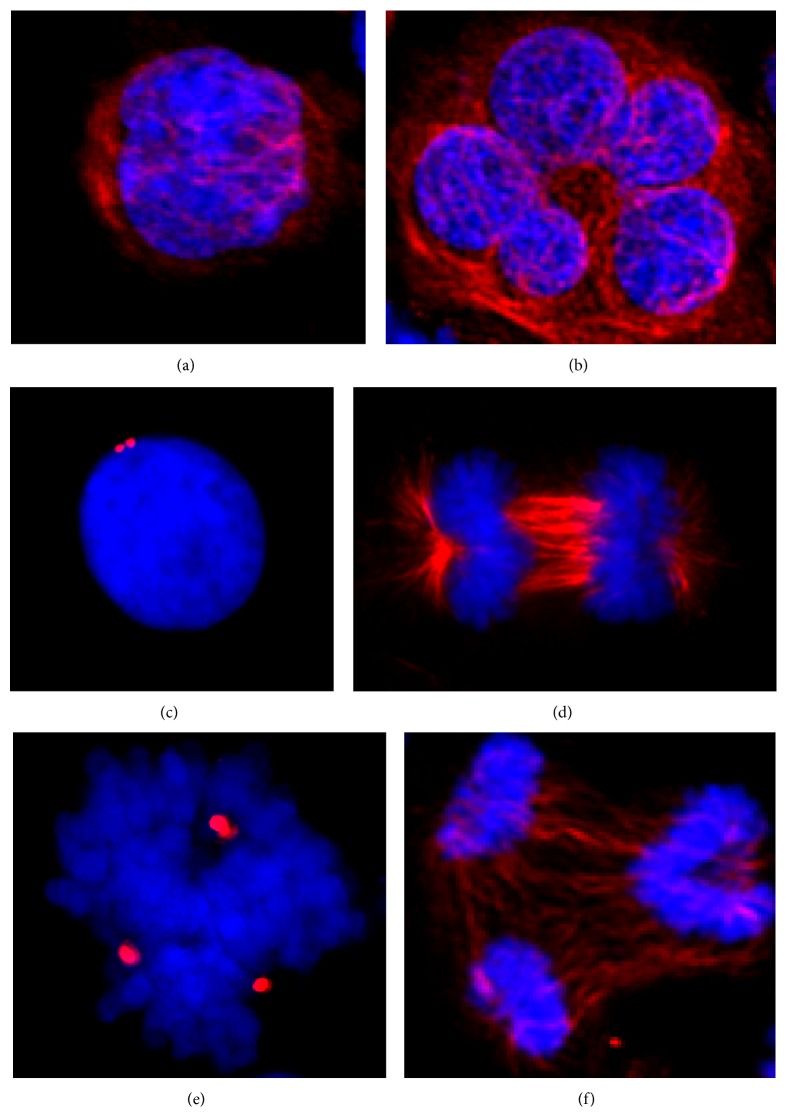
Morphological features of mitotic catastrophe. Human K562 chronic myeloid leukaemia cells during normal interphase (a) and a giant multinucleated cell following mitotic catastrophe induced by microtubule disruption (b). Interphase cell with two centrosomes (c) and normal chromosome segregation during anaphase (d). A cell containing >2 centrosomes (e) forms multipolar mitotic spindles (f) leading to aneuploidy as a result of mitotic catastrophe failure. DNA (blue), *α*-tubulin (red) (a, b, d, f), and centrosome (red pericentrin staining) (c and e).

Genome instability represents an enabling characteristic underlying the acquisition of the hallmarks of cancer [1]. Mammalian cells have evolved a variety of mechanisms to remove defective and genomically unstable cells. Mitotic catastrophe is a regulated antiproliferative process that occurs during defective or failed mitosis. Although it does not constitute a* bona fide* cell death mechanism in itself, mitotic catastrophe precedes and uses antiproliferative measures including apoptosis, necrosis, and senescence to prevent the proliferation of defective mitotic cells [2, 3]. Mitotic catastrophe is characterised by unique nuclear alterations that lead to multinucleation and/or micronucleation and are used as morphological markers for detection. Giant multinucleated cells arise from clusters of missegregated uncondensed chromosomes, whereas micronucleated cells arise from lagging chromosomes or chromosome fragments during anaphase that are left outside the daughter nuclei formed during telophase, thereby giving rise to a micronucleus in addition to the main nucleus [4].  Figure 1 illustrates the morphological features following normal cell division (a) and a multinucleated cell formed during mitotic catastrophe (b). Failure of the mitotic catastrophe antiproliferative process leads to persistent genome instability and aneuploidy (c–f). Furthermore, as a result of the various antiproliferative pathways adopted by mitotic catastrophe it is often accompanied by morphological and biochemical features of apoptosis and necrosis [2, 3].

The detection and removal of mitotically defective cells are important steps in the prevention of genome instability. Defective or failed mitosis leads to the generation of aneuploid or tetraploid cells, which are a common feature of tumour cells [5, 6]. It was postulated by Theodor Boveri more than 100 year ago that abnormality in chromosome segregation during mitosis could promote tumour formation [7]. It is now known that aneuploidy is present in approximately 90% of solid human tumours and >50% of haematopoietic cancer [8]. Some aneuploid tumours have a minor imbalance in chromosome number whereas others are characterised by a large amount of aneuploidy and contain a near tetraploid chromosome number [5]. During mitosis, the loss or gain of chromosomes can occur through a variety of mechanisms including mitotic checkpoints defects, chromosome cohesion defects that lead to sister chromatid missegregation, and centrosome amplification that promotes multipolar mitosis. The hyperstabilisation of kinetochore-microtubule attachments can also prevent the correction of previous attachment defects [5]. On the other hand, tetraploid cells have twice the normal diploid chromosome content, which can arise due to mitotic slippage, cytokinesis failure, cell fusion, and endoreplication [5]. Tetraploid cells also contain twice the normal centrosome content, which promotes multipolar mitosis and whole chromosome missegregation, and provides a mechanism for the transition of cells from a tetraploid state to an aneuploid state. Multipolar mitotic divisions generally lead to catastrophic chromosome missegregation events that are incompatible with survival; however, cancer cells can avoid such catastrophic events and suppress multipolar mitosis by clustering centrosomes into two groups thereby allowing division to occur in a bipolar fashion [9].

While there has been much debate over the role of aneuploidy and tetraploidy in tumour onset, mounting evidence suggests that tetraploid cells can trigger cellular transformation and tumour formation [5, 6]. For example, p53^−/−^ tetraploid mouse cells formed tumours when transplanted into immunocompromised mice, which was not detected with the isogenic diploid cells [10]. Tetraploid cells generated by virus induced cell-cell fusion can proliferate and induce transformation [11, 12]. Mutation of adenomatous polyposis coli (APC) in colorectal cancer resulted in tetraploid genomes* in vivo* due to cytokinesis failure [13]. Furthermore, tetraploidy was identified as an early event during cervical carcinoma [14], and tetraploid cells formed following cytokinesis failure induced transformation* in vivo* [15, 16]. In these cases transformation was coupled with extensive genome instability with abnormalities in the number and structure of chromosomes, providing evidence that tetraploidy represents an intermediate stage to promote aneuploidy and genome instability. Moreover, the loss of two tumour suppressor genes Breast Cancer Susceptibility Gene 2 (BRCA2) or the LATS1 tumour suppressor is accompanied by cytokinesis defects, suggesting a role for these tumour suppressors during cytokinesis [17, 18].

Aneuploidy increases the rate of both spontaneous and carcinogen-induced tumour formation; however, paradoxically, cases where aneuploidy does not promote tumourigenesis or where it suppresses tumourigenesis have also been reported [19]. It is clear that aneuploidy alters the path of tumour development, and a variety of factors influence the final outcome including the combination of chromosomes involved, cell type, genetic context, for example, the presence of additional cooperating mutations in key regulatory genes, as well as the microenvironment within different tissue [19]. This context driven outcome is illustrated in patients with Down syndrome who carry an extra copy of chromosome 21 and have increased incidence of haematological malignancies but reduced incidence of solid tumours [20, 21]. More recently it was suggested that the rate of chromosome missegregation will determine whether aneuploidy will promote or suppress tumour growth, where low rates of chromosome missegregation can promote tumourigenesis, and high rates lead to cell death and thereby prevent tumour growth [22]. In each scenario, the final outcome will be influenced by the functional status of damage sensing mitotic catastrophe signals as well as the cell survival and death machinery. Thus, mitotic catastrophe represents an important part of our genome maintenance machinery and abrogated or compromised signals will contribute to tumour onset. Understanding the molecular mechanism that dictates mitotic catastrophe has important implications for tumour prevention and treatment. Here we provide an update on current knowledge about the mechanism of mitotic catastrophe induction and signalling and highlight approaches to target and exploit the process in cancer treatment.

## 2. Mitosis

The cell cycle represents a highly coordinated process whereby a cell is divided into two genetically identical daughter cells. Pioneering work over the past four decades has revealed the molecular components that control the cell cycle in eukaryotes [23]. The mammalian cell cycle can be divided into distinct phases, DNA replication (Synthesis (S) phase) and division (Mitosis (M) phase), which are separated by Gap phases (G1 and G2). Mitosis is subdivided into prophase, prometaphase, metaphase, anaphase, telophase and cytokinesis, which together regulate nuclear envelope breakdown, chromosome attachment to spindle microtubules, alignment along the metaphase plate, sister chromatid separation, and finally, the coordinated plasma membrane remodelling and cytoplasmic division to produce two daughter cells [23]. Transition through the cell cycle is controlled by the interplay between cyclin-dependent kinases (cdks) and their respective cyclin binding partners [23, 24]. Activation of cdk1, which occurs upon formation of a cdk1/cyclin B complex, regulates entry and progression through mitosis. Active cdk1/cyclin B phosphorylates substrates involved in nuclear envelope breakdown, assembly of the mitotic spindle, chromosome condensation, and activation of the spindle assembly checkpoint [23, 24]. During metaphase the mitotic chromosomes, which are composed of sister chromatids held together by cohesion, are aligned on the mitotic spindle by stable microtubule attachment through their kinetochores. Properly aligned chromosomes are separated during anaphase and move towards opposite ends of the spindle [25]. A narrow region of overlapping nonkinetochore microtubules forms the central spindle at the midzone between separating chromosomes. This is followed by formation of centralspindlin comprised of MKLP1 and CYK4 and containing a GTPase-activating protein (GAP) domain, and the Chromosomal Passenger Complex (CPC) composed of Aurora B and three additional proteins, INCENP, Survivn, and Borealin that are required for Aurora B regulation [26–29]. Central spindle recruits Ect2, a RhoGTPase leading to RhoA activation and assembly of an actinomyosin contractile ring around the central core of the cell. The contractile ring constricts to form a cleavage furrow that ingresses and packs the midzone microtubules to form the dense region termed the midbody at the centre of a long intercellular bridge holding daughter cells together. During cytokinesis, the midbody acts as a platform for components required during abscission of the plasma membrane and eventual daughter cell separation [26–29].

## 3. The Spindle Assembly Checkpoint

As well as the coordinated activation and inactivation of cdk1 that controls mitotic progression, the fidelity of the process is maintained by an independent and evolutionary conserved checkpoint known as the spindle assembly checkpoint (SAC) [25]. The SAC is a surveillance process at the transition from metaphase to anaphase that monitors the attachment of chromosomes to the kinetochore spindles and halts progression of anaphase until all chromosomes are correctly attached to the bipolar spindle [25]. Upon proper attachment, the SAC is switched off and Cdc20 activates the E3 ubiquitin ligase, Anaphase Promoting Complex (APC), leading to ubiquitination and proteolytic degradation of two substrates, cyclin B, which maintains cdk1 in an active form, and securin, which inhibits separase. Following degradation of securin, the liberated separase targets cohesion causing sister chromatid separation, and, anaphase onset. Furthermore, APC-mediated degradation of cyclin B leads to inactivation of cdk1 and signals mitotic exit [25]. Thus, the SAC is active for a short time during a normal mitosis. A single unattached or incorrectly attached chromosome is sufficient to block progression to anaphase by inhibition of APC activity, thereby leading to mitotic arrest.

## 4. Mechanism of Mitotic Catastrophe

**Figure 2:**
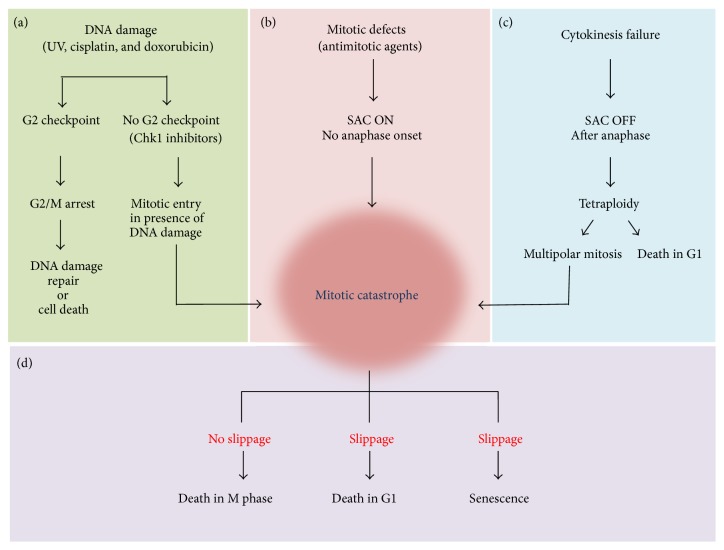
Mechanisms of mitotic catastrophe. (a) Cells with an abrogated G2 checkpoint will enter mitosis prematurely in the presence of damaged DNA and undergo segregation defects leading to mitotic catastrophe. (b) Cells with defects in mitotic apparatus and/or machinery required for faithful chromosome segregation fail to satisfy the spindle assembly checkpoint (SAC) and undergo prolonged mitotic arrest and mitotic catastrophe. (c) Cytokinesis defects that occur after anaphase will lead to a tetraploid progeny that will undergo mitotic catastrophe in the next M-phase. (d). Following activation of mitotic catastrophe, cells arrested in mitosis have three fates; they will undergo death in mitosis in the presence of cyclin B, or cyclin B levels will gradually fall allowing the cells to undergo slippage and exit mitosis where they subsequently undergo death in G1. Alternatively, cells can undergo senescence following slippage.

Mitotic catastrophe senses mitotic damage and directs the defective cell to one of three possible antiproliferative fates (Figure 2). Defective mitotic cells can engage the cell death machinery and undergo death in mitosis, when cyclin B levels remain high. Alternatively, defective cells can exit mitosis, known as slippage, and undergo cell death execution during G1 in the subsequent cell cycle. Finally, defective cells can exit mitosis and undergo senescence [2, 3]. It is clear that mitotic catastrophe is always accompanied by mitotic arrest; however, the mechanisms that dictate cell fate following mitotic catastrophe remain unclear [2, 3]. It was originally proposed that death signals gradually accumulate during mitotic arrest, and therefore the length of mitotic arrest determines cell fate [30]. Since then a model has been proposed whereby cell fate is dictated by two independent, yet competing networks; one involves activation of prodeath signals and the other protects against cyclin B degradation. Both pathways work in opposite directions during prolonged mitosis; that is, cell death signals accumulate and cyclin B levels decline. Both pathways have a threshold and the fate of the cell is determined by which threshold is breached first [31]. It is known that cyclin B levels slowly decline during prolonged mitotic arrest even in presence of an active SAC [32]; thus, if levels fall below the threshold that dictates mitotic exit, slippage occurs, whereas if the death threshold is met first the cell will undergo death in mitosis. Additional work has focused on determining the molecular events that govern each network and its threshold in order to understand how cells respond to mitotic stress [33].

The activity of the Bcl-2 protein family is a key determinant of fate following mitotic catastrophe [67–71] and phosphorylation mediated by cdk1 is an important signal that controls Bcl-2 family activity [67, 68, 72, 73]. The family is comprised of multidomain prosurvival proteins (Bcl-2, Bcl-_XL_, Bcl-_W_, Mcl1, A1, and Bcl-B) and multidomain proapoptotic effector proteins (Bax, Bak, and Bok) as well as BH3-only proteins (Bim, PUMA, Bad, NOXA, Bik, Hrk, Bmf, and tBid) [74]. The multidomain members of the family (the prosurvival proteins and the effectors Bak, Bax, and Bok) contain four BCL-2 homology regions (BH1–BH4), whereas the BH3-only proteins contain only a BH3 domain, which is important in mediating their interaction with the multidomain members. Various models are proposed to describe how prosurvival and proapoptotic Bcl-2 proteins interact together to control apoptosis. For a recent review see [74].

Bcl-2 proteins are also regulated in a transcriptional and posttranslational manner. Active cyclin B/cdk1 directly phosphorylates Bcl-2, Bcl-_XL_, and Mcl-1 during mitosis and negatively regulates their activity [67, 68]. Cdk1 phosphorylation of Bcl-2 and Bcl-_XL_ blocks heterodimer formation with proapoptotic members, Bax and Bak, promoting their oligomerization at the outer mitochondrial membrane, release of cytochrome C, and thereby apoptosis [75, 76]. In contrast, cdk1-mediated phosphorylation of Mcl-1 during mitosis controls protein stability by ubiquitination and degradation via the proteasome. Harley et al. [68] demonstrated that phosphorylation of Mcl-1 by cdk1/cyclin B initiates its degradation during mitotic arrest in a Cdc20/APC-3 dependent manner. Like Bcl-2 and Bcl-_XL_, loss of antiapoptotic Mcl-1 promotes the oligomerisation of Bax and Bak and thus death during prolonged mitotic arrest. Thus, it is proposed that during a typical mitosis the transient phosphorylation of Mcl-1 by cdk1/cyclin B is not sufficient to drive cell death before cyclin B levels drop sufficiently to inactive cdk1. This transient effect ensures that normal cells are not subject to a fate of apoptosis during normal mitosis. In contrast, however, the sustained cdk1 activity that occurs during mitotic arrest leads to a significant drop in Mcl-1 levels thereby suppressing its antiapoptotic effect and triggering cell death before mitotic exit. Phosphorylation of Mcl-1 also controls interaction with the FBW7 tumour-suppressor and subsequent degradation by the skp-cullin-F-box (SCF) complex during mitotic arrest [77]. Collectively, these reports highlight a role for posttranslational phosphorylation and ubiquitination in the crosstalk between the mitotic and apoptotic machinery to control cell fate during mitotic arrest.

The BH3-only protein Bim undergoes cdk-1-mediated phosphorylation during mitosis. Mac Fhearraigh and Mc Gee [72] demonstrated that two Bim isoforms, Bim_EL_ and Bim_L_, undergo transient phosphorylation during normal mitosis; however, hyperphosphorylation was evident during sustained mitotic arrest. Furthermore, Bim directly binds cyclin B, which acts as a molecular bridge for cdk1 phosphorylation, and serine 44 within Bim_L_ was identified as a novel cdk-1 phosphorylation site [72]. It is suggested that cdk-1-mediated phosphorylation of Bim alters its heterodimer formation with Bcl-2, leading to enhanced activation of Bak and mitochondrial cell death [75], consistent with the view that mitochondrial proapoptotic signalling entails the interplay between pro- and antiapoptotic Bcl-2 proteins [78]. In contrast, cdk1-dependent phosphorylation of Bim_EL_ promotes its polyubiquitylation and degradation via the proteasome during mitotic arrest [73]. Thus, based on the competing model that was proposed by Gascoigne and Taylor, [31] the gradual loss of cyclin B during prolonged mitotic arrest will lead to the loss of cdk-1-mediated Bim phosphorylation, which will alter its cell death activity during prolonged mitosis and following slippage, either through stability or heterodimer formation. This may partially explain the contradictory reports that mitotic catastrophe-induced death occurs via Bim-dependent and Bim-independent mechanisms [79–82].

Cdk1 also phosphorylates members of the proteolytic caspase family, specifically caspase-2, caspase-8, and caspase-9, leading to inhibition of their apoptotic activity, which is believed to be a cytoprotective measure during normal mitosis [83–85]. Furthermore, caspase activity is not required for spindle assembly checkpoint function or mitotic slippage following mitotic catastrophe [86]; however, the downstream cell death can manifest in a caspase-dependent or caspase-independent manner [3]. Cell death can involve mitochondrial perturbations including mitochondrial outer membrane permeabilisation (MOMP) and cytochrome C release induced following Bax/Bak oligomerisation and pore formation on the outer mitochondrial membrane, leading to caspase-dependent apoptosis [87]. Alternatively cell death can occur through the Permeability Transition Pore Complex (PTPC), a large complex that bridges the junction between the inner and outer mitochondrial membranes. A sudden increase in permeability of the inner mitochondrial membrane to small solutes leads to Ca^2+^ overload, oxidative stress, and Mitochondrial Permeability Transition- (MPT-) induced death that is independent of caspase activity [88]. Although details of the mitotic and cell death processes are well characterised, the molecular signals that link these events during mitotic catastrophe remain poorly understood and are the focus of intense investigation. Two interesting candidates are Mad2 and survivin that are reported to have dual functions in regulating spindle checkpoint and cell death [58, 89]. Furthermore, it was recently shown that mitochondrial Protein Tyrosine Phosphatase 1B (PTP1B) undergoes coordinate phosphorylation by cdk1 and plk1 during mitotic arrest. Phosphorylation of mitochondrial PTP1B increases its phosphatase activity and sensitises cells to antimitotic agents [90] representing a new molecular link between the mitotic machinery and the mitochondrion during mitotic catastrophe. The identification of PTP1B substrates at the mitochondria will provide better insight into its precise function and help delineate the downstream execution events. It was also recently demonstrated that cdk1 can directly phosphorylate and regulate numerous mitochondrial proteins, including subunits of the respiratory chain to regulate respiration in a cell cycle specific manner [91, 92], providing a further link between cdk1 activity and mitochondrial function. It remains to be determined how mitochondrial energetics are altered during mitotic catastrophe.

## 5. Induction of Mitotic Catastrophe by Mitotic Perturbations

Faithful mitotic progression requires the proper function of various cell components including microtubules, mitotic enzymes, motor proteins, and protein complexes [34, 93]. Microtubules are essential cytoskeletal components composed of subunits of *α*-tubulin and *β*-tubulin that dimerise to form linear protofilaments, which together form microtubules. The dynamic nature of microtubule plus ends, which undergo continuous polymerisation and depolymerisation, allow them to form cell structure and enable motility and intracellular transport [93]. During mitosis microtubules form a bipolar spindle array that emanates from the centrosomes located at opposite sides of the cell. The dynamic nature of microtubule ends facilitates proper attachment to chromosomes at their kinetochore [25]. Unattached or incorrectly attached kinetochores, such as merotelic or syntelic attachments, initiate a network of signals to recruit mitotic checkpoint components including Mad1, Mad2, Bub1, Bub3, BubR1, CENP-E, and Mps1 to kinetochores [25, 94–96]. The formation of an inhibitory complex termed the mitotic checkpoint complex (MCC), consisting of three SAC components, Mad2, BubR1, and Bub3 as well as Cdc20, acts as the SAC effector that is enriched at unattached kinetochores. MCC binds to and potently inhibits APC by sequestering Cdc20, thereby preventing mitotic exit [25, 94–96] (Figure 2). Biorientated attachment of all sister chromatid pairs to their kinetochore microtubules promotes displacement of the SAC proteins, allowing release of Cdc20 from the MCC. Released Cdc20 can then activate the APC and promote mitotic exit. Improper kinetochore-microtubule attachment also causes reduced tension across the spindle apparatus which inhibits the APC through a mechanism involving Aurora B kinase [97, 98].

Most cancer cells display a certain level of aneuploidy [5, 6, 19], and it was proposed that mechanisms that induce additional instability constitute a therapeutic strategy. Consistent with that, cancer cells are more susceptible to cell death following mitotic damage in comparison to nontransformed cells [99], and a number of mitotic targets have been identified. These include mitotic kinases such as aurora kinases, monopolar spindle 1 (Mps1), and polo-like kinases (Plks) that play key roles during faithful chromosome segregation [100]. The aurora kinase family of serine/threonine protein kinases includes Aurora A, Aurora B and Aurora C, each with a distinct expression pattern, subcellular localisation pattern, and function [101, 102]. Aurora A localises to centrosomes during interphase and to spindles poles and spindle microtubules during mitosis, where it regulates mitotic entry, centrosome maturation, and spindle formation [35]. Aurora B localises to kinetochores and forms part of the Chromosome Passenger Complex that plays critical roles during chromosome condensation, biorientation, and cytokinesis [97]. Thus, Aurora A and Aurora B act at different stages of mitosis. Aurora C is mainly expressed in testes and required for spermatogenesis and mouse embryogenesis [35]. Dysregulated aurora kinase activity generates mitotic abnormalities and cytokinesis failure [103, 104]. Thus, the critical role of aurora kinases during mitosis makes them indispensible with faithful mitosis.

Mps1 forms a core component of the spindle assembly checkpoint (SAC) and functions in the alignment and orientation of chromosomes during metaphase [105]. Polo-like kinases (Plks) also play critical roles during mitotic progression [102, 106]. Five members of the Plk family have been identified in humans. The most widely studied is Plk1, which is involved in assembly of the mitotic spindle, maturation of centrosomes, activation of the SAC, chromosome segregation, and cytokinesis [102, 106]. Deregulation of the centrosome cycle leading to supernumerary centrosomes generates multipolar mitosis that promotes genome instability, SAC activation, and mitotic catastrophe [107, 108].

Faithful mitosis is also dependent on microtubule motor proteins such as Eg5, a plus-end directed motor from the kinesin superfamily that is responsible for mitotic spindle formation and function. Disruption of Eg5 function during mitosis leads to monopolar spindles and activation of the SAC. Furthermore, the centromere-associated motor protein (CENP-E) is a component of the kinetochore corona fibres of mammalian centromeres and is required for chromosome biorientated attachment and proper mitotic checkpoint signalling [109–111]. In addition to disrupting chromosome segregation, inhibition of cytoplasmic division following anaphase onset will generate genome instability and stimulate mitotic catastrophe in the next cell cycle [109, 112] (Figure 2). The development of pharmacological agents that induce mitotic catastrophe via disruption of bipolar spindle function or faithful chromosome segregation is discussed in more details later.

## 6. Induction of Mitotic Catastrophe following DNA Damage

DNA damage induced by intrinsic or extrinsic factors threatens genome integrity and stability. Failure to repair the DNA damage leads to mutations and genome instability that ultimately contributes to diseases including cancer [113, 114]. The DNA Damage Response (DDR) is a complex mechanism to sense various types of DNA damage and respond appropriately to maintain genomic integrity. Following DNA damage the phosphatidylinositol 3-kinase-related kinases ATM (ataxia-telangiectasia mutated) and ATR (ATM and Rad-3 related) are activated and coordinate activation of the DNA damage checkpoints through the phosphorylation of numerous downstream substrates. Checkpoint kinase-1 (Chk1) and checkpoint kinase-2 (Chk2) are serine threonine kinases that transduce the DNA damage signal downstream. Chk2, which is expressed throughout the cell cycle, undergoes phosphorylation and activation by ATM, whereas Chk1 is preferentially expressed in S and G2 and is phosphorylated by ATR. In addition to Chk1 and Chk2, MAPK-activated protein kinase-2 (MK2) regulates cell cycle checkpoint activation [113, 114]. Genomic stress activates the G1 checkpoint, which prevents S phase entry by inhibition of DNA replication. At this point, Chk2, which is activated by ATM, phosphorylates and suppresses the phosphatase Cdc25-A, thereby preventing activation of cyclin E/cdk2 and thus halting the cell cycle. The S phase checkpoint is activated in response to replication errors and DNA damage that occurs during S phase, whereas the G2 checkpoint deals with cells that have either undergone DNA damage in G2, or they have escaped the G1 and S phase checkpoints. Cdk1 activity and mitotic entry are tightly regulated and balanced by inactivating phosphorylation by the protein kinases WEE1 and myelin transcription factor 1 (MYT1), together with the activating Cdc25 phosphatase. Thus, WEE1 and Cdc25 act as a central switch for mitosis and are regulated by posttranslational alterations thereby enabling rapid switching. At G2, Chk1, which is activated by ATR, phosphorylates and suppresses Cdc25-A, -B, and -C thereby preventing cyclin B/cdk1 activation and causing G2 arrest [113–115]. G2 arrest is also initiated by MK2 which inactivates Cdc25-B and -C [113]. Thus, the G2 checkpoint is the last opportunity to halt the cycle and repair DNA damage in cells that have escaped the G1 and S phase checkpoints. Abrogated or compromised G2 checkpoint will allow premature mitotic entry of defective cells that fail to undergo proper chromosome segregation thereby leading to mitotic catastrophe (Figure 2). In support of this, the fusion of interphase and mitotic cells led to mitotic catastrophe which was due to the cyclin B/cdk1 driven-premature entry of cells into mitosis before they had completed S or G2 [116]. Furthermore, knockout of the cytoplasmic binding protein 14-3-3*σ* in colorectal cancer cells resulted in failure to sequester cyclin B1 and prevented G2 arrest following DNA damage, culminating in mitotic catastrophe [117]. Inhibition of Chk2 also abrogates the G2 checkpoint leading to mitotic catastrophe following DNA damage. [118]. In contrast, cells that harbour DNA damage and undergo death in interphase do not constitute an example of mitotic catastrophe [2, 3, 113]. Furthermore, while an abrogated or defective G2 checkpoint is essential for DNA damage-induced mitotic catastrophe, the eventual mode of cell death induced is determined by whether p53 is present or absent. For example, DNA damage induces two distinct forms of cell death in ovarian carcinoma [119]. Functional p53 triggered apoptosis in ovarian carcinoma cells following mitotic catastrophe whereas loss of p53 in these cells triggered necrosis. The exact mechanism of p53 activation during or after mitotic catastrophe remains to be elucidated; however, it was shown that phosphorylated H2AX-ATM-p53 pathway dictates an apoptotic outcome following mitotic catastrophe. Loss of p53 or depletion of ATM protected against apoptosis and instead led to necrosis [4]. Apoptosis driven by p53 is also associated with caspase activity [120]. Thus it is proposed that the initiation of mitotic catastrophe occurs independently of p53 status and caspase activity; however, the presence of functional p53 is required for a caspase-mediated apoptotic response.

## 7. Exploiting Mitotic Catastrophe in Cancer Therapy

Mitotic catastrophe is induced by a variety of agents classified as those that disrupt mitotic progression or directly damage DNA (Figure 2 and Table 1). The best known antimitotic agents are the microtubule targeting agents (MTAs), also known as spindle poisons [34]. MTAs are grouped into two families: the microtubule polymerisers which include the taxanes (paclitaxel and docetaxel) and the microtubule depolymerisers which include the vinca alkaloids (vinblastine and vincristine). The suppression of microtubule dynamics by both groups precludes normal bipolar spindle formation and prevents chromosome biorientation, leading to mitotic arrest and cell death. Taxol (paclitaxel) originally isolated in 1967 from a Yew tree (*Taxus brevifolia*), was approved for clinical use in 1995 and is widely used across a range of malignancies. For example, taxanes have been used in the treatment of Kaposi's sarcoma, non-small-cell lung cancer, breast cancer, ovarian cancer, and prostate cancer, whereas vinca alkaloids are used to treat haematological malignancies [34, 121]. Although they have been used clinically for decades, microtubule targeting agents lack specificity towards cancer cells and disrupt other important microtubule-dependent functions leading to severe side effects including neuropathy. Furthermore, the development of drug resistance limits their use, which can be ascribed to drug efflux pumps, overexpression of prosurvival Bcl-2 proteins, and mutations in tubulin that abrogates drug binding [122, 123]. Resistance may also occur as a result of mitotic slippage [32]. Thus, research efforts have focused on the development of non-microtubule antimitotic therapeutics, such as those targeted at mitotic kinases and spindle motor proteins, with the hope that that they would overcome some of the drawbacks associated with microtubule targeting agents.

Primary tumours frequently have overexpressed and/or amplified aurora kinases. Moreover, their depletion or inhibition impairs the proliferation of cancer cells, thus, making them an attractive target for cancer treatment [35, 36]. A number of aurora kinase inhibitors have been developed that target the enzymes ATP binding domain. Early inhibitors did not display specificity towards a family member; however, in recent years work has focused on development of selective inhibitors and a number are in various stages of clinical evaluation including Alisertib (MLN8237) that has displayed promising antitumour properties and is currently in Phases I and II trials [35].

Mps1 is highly expressed in human tumours where it promotes cell proliferation [48]. Mps1 kinase inhibitors have been developed which induce mitotic defects and death in cancer cells, across a variety of preclinical models, either alone or in combination with microtubule inhibitors [49–51].

Plk1 is upregulated in a range of human tumours; thus targeting Plk1 is an attractive therapeutic strategy [45] and a number of inhibitors are under clinical evaluation including BI2536 [46, 47]. Furthermore, agents that disrupt the centrosome cycle in tumour cells, through centrosome amplification and centrosome declustering promote multipolar mitosis, genome instability, and mitotic catastrophe [55–57].

A number of kinesin motor protein inhibitors have been developed [37, 124]. Monastrol is a selective inhibitor of the kinesin-5 motor protein (KSP, also termed Eg5). Eg5 inhibition leads to mitotic arrest and death in tumour cells in culture and in xenograft models. Furthermore, they were found to be free from severe cytotoxic effects and are generally well tolerated [37]. Eg5 inhibitors under clinical development include AZD4877 [38], Ispinesib [39, 40], and ARRY-520 [41]. Eg5 inhibition is also effective in targeting taxol resistant cancers [42]. CENP-E (centrosome-associated protein-E) is a microtubule motor that plays a role in mitosis. The small molecule CENP-E inhibitor, GSK923295, induces defective mitosis and displays antiproliferative effects* in vivo* [43] and has recently entered clinical trial [44].

Cancer cells often harbour a deficient or defective G1 checkpoint due to aberrant p53 signalling, which ultimately leads to increased DNA damage at the G2 checkpoint compared to normal cells [125]. Based on this, the G2 checkpoint has emerged as an attractive anticancer target. Abrogation of the G2 checkpoint allows cells with unrepaired DNA damage to proceed into a premature M phase [59]. Thus, cancer cells that are defective in G1 and G2 checkpoints will undergo mitotic catastrophe following DNA damage induced by radiation, alkylating agents, and doxorubicin [113]. The Chk1 kinase inhibitors, UCN-01 and AZD7762, abrogate the G2 checkpoint and potentiate death in p53-deficient tumours [58, 60, 61] and are currently in clinical development [62, 63]. Evaluation of a panel of therapeutic agents in combination with Chk1 inhibition highlights that the precise drug combinations are important and influence the outcome in a particular genetic background and when treating a certain tumour type [126]. In addition to Chk1, targeting WEE1 kinase activity together with DNA damage can effectively induce mitotic catastrophe [127]. Furthermore, histone deacetylase (HDAC) inhibitors promote mitotic catastrophe and cell death and have shown promise in multiple myeloma and glioma treatments [64–66].

Despite the promising preclinical data displayed by new generation antimitotic agents, their clinical efficacy has been disappointing in comparison to microtubule targeting agents [37, 128]. This may be explained by the shorter doubling time of cells in culture compared to patients [128] and by differences in drug retention times [129]. It has also been suggested that the success of microtubule targeting agents may be due to nonmitotic function of microtubules [130]. Furthermore, the development of resistance to antimitotic agents represents a major challenge that occurs following mitotic slippage when defective cells adapt and survive [131], although in some cases mitotic slippage is required for cell death [132]. Based on this observation, recent research has focused on strategies to block slippage and mitotic exit in order to maximise mitotic arrest-induced death [37]. Such approaches include targeting APC-Cdc20 to prevent cyclin B degradation and mitotic exit that have shown a very promising response [52–54]. An alternative approach is the inhibition of cytokinesis which blocks mitotic exit in postanaphase cells and may be an attractive strategy to overcome resistance in slippage prone cells. Small molecule dynamin GTPase inhibitors have shown antiproliferative effects and induce cytokinesis failure and cell death in cancer cells [133].

It is clear that mitotic catastrophe is an important anticancer strategy that is achieved by a variety of mechanisms that target the cell cycle. Although these approaches target proteins that are upregulated in cancer cells, thereby providing a therapeutic window to preferentially kill the cancer cells, they are not specific to cancer cells and are likely to be accompanied by some side effects. Recent reports highlight that the myc oncogene regulates mitotic events to support its oncogenic program [134, 135] and one way that this may occur is through transcriptional regulation of aurora kinase expression [136, 137]. Moreover, loss of myc activity due to inhibition of sumoylation [134] or transcriptional inactivation by omomyc [135] led to mitotic catastrophe and cell death in* in vivo* models of breast cancer and glioma, respectively. Thus, targeting myc activity, using approaches that inhibit sumoylation and/or mimic omomyc action, represents new approaches to selectively induce mitotic catastrophe in cancer cells. Furthermore, a better understanding of the postmitotic signals that connect to the cell death and senescence pathways will reveal new approaches to push cells down a defined antiproliferative route and is likely to synergise with current antimitotic drugs to kill cancer cells before adaption and the development of drug resistance. These new approaches may provide more effective strategies to exploit mitotic catastrophe in cancer prevention and treatment.

## Figures and Tables

**Table 1:** Exploiting mitotic catastrophe in cancer therapy.

Mechanism of action	Inducer	References
*Microtubule targeting agents *		
Microtubule polymerisers	Taxanes	[34]
Microtubule depolymerisers	Vinca alkaloids	

*Non-microtubule antimitotic agents *		
Mitotic spindle targets	Aurora kinase inhibitors *Alisertib *	[35, 36]
	KSP inhibitors *Eg5* *AZD4877* *Ispinesib* *ARRY-520 *	[37–42]
	CENP-E inhibitors *GSK923295 *	[43, 44]
	PLK-1 inhibitors *B12536 *	[45–47]
Mitotic checkpoint targets	MPS1 inhibitors *NMS-P715* *MPS1-IN-3 *	[48–51]
Mitotic exit inhibition	APC inhibitor *TAME *	[52–54]
Centrosome disruption	*Griseofulvin *	[55–57]

G2 checkpoint abrogation	Chk1 inhibitors *UCN-01* *AZD7762 *	[58–63]
	HDAC inhibition *Trichostatin A *	[64–66]
